# The acceptability of nicotine containing products as alternatives to cigarettes: findings from two pilot studies

**DOI:** 10.1186/1477-7517-8-27

**Published:** 2011-10-12

**Authors:** Ron Borland, Lin Li, Kevin Mortimer, Ann McNeil, Bill King, Richard J O'Connor

**Affiliations:** 1 VicHealth Center for Tobacco Control, The Cancer Council Victoria, Carlton 3053, Australia; 2 Epidemiology and Public Health, University of Nottingham, Nottingham, UK; 3 Department of Health Behavior, Roswell Park Cancer Institute, Buffalo, NY, USA

## Abstract

**Background:**

This study aimed to explore issues that might impact on the acceptability and feasibility of offering smokers nicotine containing products either to quit nicotine use altogether by using as a short term means of quitting cigarettes or as a longer term substitute.

**Method:**

Two small pilot studies, one in the UK (n = 34) involving face to face contact and direct provision of the product, the other in Australia (n = 31) conducted remotely with products sent in the mail.

**Results:**

Nicotine lozenges were the most popular products, but significant minorities liked a smokeless product more. Use stimulated interest in quitting, and although many failed to use all the products provided, most were interested in future use, more often to help quit than as a planned long-term substitute.

**Conclusions:**

These studies indicate an untapped interest in the use of substitutes to reduce the harmfulness of smoking. Studies of this sort do not inhibit interest in quitting nicotine altogether, and may facilitate it. The greater the range of products on offer, the more smokers are likely to try a product to quit.

## Background

Harm reduction, or encouraging cigarette smokers to move toward less hazardous forms of nicotine delivery, is a potential means to reduce the overall morbidity and mortality associated with smoking. Martin et al (2004) identified 5 characteristics harm reducing products should have: substantial disease reduction, minimal unintended (adverse) consequences, no combustion and large reduction in toxins, acceptable to consumers, and documented scientific basis for harm reduction [1]. Nicotine replacement therapy (NRT) products are the first obvious potential substitutes, however, there are concerns about consumer acceptability for long-term use [1]. While some forms of smokeless tobacco are quite toxic, others have toxicant profiles similar to that of NRT, so an alternative strategy would be to consider the lower toxicant versions of smokeless tobacco, if NRT was not acceptable to them. The viability of these strategies hinges in part on how likely cigarette smokers would be to use them, how much reduction in harm we might expect, and whether introducing such products would prolong tobacco use or even encourage new use, and the likely net effects of these forces.

If substitution was to be a viable strategy, smokers would need to be convinced to try such products and to use them for long enough to establish whether they were an adequate substitute for cigarettes. There are an increasing number of small studies testing smokers' willingness to switch to smokeless or long term use of NRT [2-5]. Rennard et al reported modest continued participation and low rates of quitting cigarettes in a year long study of use of the nicotine inhaler to reduce or enable quitting of smoking [6]. The 20% who continued inhaler use did manage to sustain large reductions in cigarette consumption. This is consistent with the view that current NRT products may not be sufficiently attractive to wean most smokers off cigarettes, but that at least some smokers will persist in using a partial substitute. O'Connor and colleagues showed that US smokers offered the opportunity to sample a range of oral nicotine products used them primarily for partial substitution (i.e. they continued to smoke, but fewer cigarettes per day) [5]. More smokers in this study preferred a nicotine replacement product than a tobacco-based product.

In a companion paper (Borland R, Li L, Cummings KM, O'Connor R, Mortimer K, & Wikmans T, McNeill A, King B: Effects of a fact sheet on beliefs about the harmfulness of alternative nicotine delivery systems compared with cigarettes. Submitted)., we showed that providing information to smokers (including the samples studied here) increased their understanding of the greater harms of cigarettes compared to both smokeless tobacco (ST) and NRT products. We believed it is essential to provide this information in studies of such products to help smokers understand why health-oriented researchers would be interested in getting them to try them for potential use.

This paper reports on two pilot studies designed to assess the feasibility of offering smokers alternative nicotine products to consider for use to quit smoking or as a long-term substitute for cigarettes. The aim is to assess preparedness to use alternatives, both short term and longer term. Subsidiary aims are to test out methods and to undertake preliminary assessment of the promise of various NRT and ST products, and to obtain estimates of possible effect sizes for powering a proposed comprehensive test of the likely outcomes of promoting these products in preference to cigarettes (and other smoked products).

## Methods

### Participants

The two studies recruited smokers in very different ways. In the UK respondents were interviewed face to face after responding to a newspaper advertisement seeking adult smokers interested in a study on alternatives to cigarettes. In Australia, the entire study was done by telephone and internet, with respondents to an anonymous survey about the harms of smoking invited into this study if they met the inclusion criteria. Participants then needed to identify themselves and return a signed consent form.

Inclusion criteria for both studies were being adults, daily smokers of 10 or more cigarettes per day, not planning to quit smoking in the next month, and reporting no regular use of medication for mental health issues. Participants also had to complete two preliminary surveys on knowledge and attitudes between which they were given a fact sheet explaining the relative harms of nicotine-containing products including cigarettes and the mechanisms by which cigarettes and other nicotine products had their harmful effects (see Borland et al, submitted, for details).

Characteristics of both samples can be found in Table 1. In the UK 43 of 77 eligible smokers accepted the offer to participate and consented and 34 (79%) tried at least one product and completed the final questionnaire.

**Table 1:** Demographic and smoking-related characteristics of the samples

Characteristics	UK (n = 34)	Australia (n = 31)
Males, n (% of total)	58.8%	51.6%
Mean Age ± SD, years	44.2 (12.2)	42.7 (13.7)
Percent with tertiary education	11.8%	35.5%
Cigarettes smoked per day, n (% of total)		
10-15	44.1%	45.2%
16-20	20.6%	16.1%
21+	35.3%	38.7%
Time (mins) between waking and 1^st ^cigarette, n (% of total)		
< = 5	9 (29.0)	12 (38.7)
6-30	16 (51.6)	13 (42.0)
> = 31	6 (19.4)	6 (19.3)
Self-perceived addiction, n (% of total)		
Very addicted	73.5	87.1

Note: Numbers given where missing data.

Of those eligible to participate (and invited), 36 of 62 in Australia accepted the offer and consented, and of these, 31 participants tried at least one product and started the post-use survey, however only 29 completed it (due to a computer problem).

In both countries the participants were heavier smokers (in part due to only selecting 10 plus per day smokers).

### Design

In both countries participants who had previously completed two surveys about beliefs about alternatives to cigarettes and had read a fact sheet about the issue were provided with product samples: In the UK participants were provided with a box with 36 nicotine lozenges (NiQuitin CQ^® ^4 mg) and a box with 30 pieces of Oliver Twist. Oliver Twist can be purchased legally in England, although it is not widely available. In Australia it was 3 of 4 possible products (Nicotine lozenge (Nicobate), 2 sizes of teabags of Swedish Snus (Catch), tobacco bits (Oliver Twist), or compressed tobacco tablets (Stonewall and/or Ariva)), with enough of each to last at least 5 days. More dependent smokers were given more of the stronger form of the two 2-strength options (2 strengths of Snus, and Ariva/Stonewall), and those less dependent more of the weaker form. The three ST products cannot be purchased in Australia, but can be legally imported for personal use.

In both countries participants were asked to look at the products and to use them only if they wanted to, either to cut down on their current level of smoking or to try to quit.

In Australia, participants were telephoned around one week after the products were sent to ensure the person had received them, to check about any issues they had, and to get feedback on initial reactions.

### Ethical approval

The UK study was approved by Nottingham Research Ethics Committee 2 and the Australian one by the Cancer Council Victoria Ethics Committee. In both countries written informed consent was obtained from all subjects prior to being sent/given any of the products.

## Results

### UK Study

In the UK study, all 34 participants who returned the final questionnaire tried at least some of the products provided (one did not try the ST). Results are summarised in Table 2. Only a minority used the entire supply. Overall, 44% (15/34) preferred the NRT lozenges, 27% (9) preferred the ST pieces; one person liked both equally (3%), but 24% (8) liked neither product. Views were broadly divided about how suitable lozenges and tobacco pieces would be as longer-term substitutes for smoking but sizeable proportions of participants thought the lozenges and tobacco pieces were possibly good enough or good enough to replace smoking (71% (24/34) and 44% (15) respectively), or 79% (27) at least one, only a small increase for adding the ST (not in Table 2).

**Table 2:** Reactions to the products and beliefs about use for each country

Question asked post use	UK (Total n = 34)	Australia (Total n = 29)
Likelihood of using an NRT product for next quit attempt		
Very likely	44.1%	53.8%
Likely	29.4%	7.7%
Uncertain	5.9%	26.9%
Unlikely/Very unlikely	17.6%	11.5%
Not answered	2.9%	-
Not asked	-	3 (all quit)
Likelihood of using an NRT product as a substitute for longer term		
Definitely would (yes seriously)	38.2%	20.7%
Probably would(yes but not seriously)	29.4%	13.8%
Possibly would	17.7%	24.1%
Probably not(no)	8.8%	27.6%
Certainly not	5.9%	13.8%
Likelihood of using ST to quit smoking if available		
Very likely	29.4%	42.3%
Likely	32.4%	26.9%
Uncertain	11.8%	19.2%
Unlikely	17.7%	7.7%
Very unlikely	8.8%	3.8%
Not asked	-	3 (all quit)
Support for law reducing cig availability		
Strongly oppose such a law	14.7%	6.9%
Probably oppose	14.7%	17.2%
I don't have a view either way	29.4%	24.1%
Probably support	35.3%	37.9%
Strongly support such a law	5.9%	13.8%
Intention to quit, n (% of total valid cases)		
No intention to quit	5.9%	0%
Open to the possibility of quitting	58.8%	30.8%
Thinking of quitting, but not in the next month	11.8%	26.9%
Planning to quit in the next month	8.8%	42.3%
Already quit	8.8%	11.5%

When asked what they would be most likely to do if ST were available on the market in the UK, 61.8% (21/34) indicated they were likely to use it. Just over half (53% or 18) indicated it was likely that they would consider ST for long term use if they were unable to quit smoking without it.

There was no clear evidence of any systematic shifts in respondents' knowledge or interest in using the products from before to after use.

Three of the 34 participants reported that they quit smoking during the experience of use sub-study. Two said the products supplied had helped them to quit; both preferred the NRT over the ST. The third participant who quit preferred the ST but did not use it to aid their quit attempt. Another 3 were planning to quit in the next month.

When asked at the end of the questionnaire if they would support laws reducing cigarette availability 29.4% (10/34) said they would not and 41.2% (14) said they probably or definitely would. Those answering anything other than definitely YES, were asked if they would be more likely to support the law if substitutes were widely available and only 20% (2/10) opposed changed their beliefs at all, while 82% (18/22) without an opinion or probably supportive becoming more supportive.

### Australian study

In Australia, of the 31 participants, 17 said they tried all 3 products provided, three tried 2, and the remaining 11 only tried one. Few respondents had used all of the supplied products when resurveyed, and substantial minorities of those given tobacco bits (31%, 5/16) and tobacco tablets (39%, 6/15) had not yet tried them. When those 17 trying all three products they were sent were asked which product they enjoyed using most, the most commonly cited products were nicotine lozenges (by 5) and tobacco bits (Oliver Twist, by 6), whereas only 2 cited tobacco "teabags" (Snus) and 1 cited tobacco tablets (Stonewall/Ariva). Three said they did not like any of them.

Views of the individual products varied considerably. For the 17 who specifically reported on Lozenge use, 8 thought it an adequate substitute (good enough, although for 6 not as good as smoking), and another 3 possibly good enough. For the smokeless tobacco products views were typically more negative. Of 16 reporting on the tobacco bits, 4 indicated they were good enough (2 unqualified) and another 3 possibly good enough. Of the 15 reporting on the tobacco tablets 6 indicated good enough (1 unqualified), and 6 more possibly good enough, and for the 14 reporting on the teabags, 3 thought them good enough and another 2 possibly so. Overall, 72.4% (21/29) thought at least one possibly good enough (including 38% (11) good enough, but not as good as smoking, and 14% (4) at least as good) and of the remainder only 14% (4) said it was definitely not good enough.

Three of the 31 participants reported that they were not smoking at the time of the final survey. One participant who used all nicotine lozenges and tobacco tablets and tried some Oliver Twist reported she was not smoking at the final survey. She said the products supplied had helped her to quit. Unlike in the UK, these respondents (quitters) were not asked about use on subsequent quit attempts. Of the remainder, 61.5% (16/26) said they were likely to use a NRT product on their next quit attempt, and 34.5% (9) said they probably would use as a long term substitute if necessary. In response to a more general question on use of ST for a quit attempt, 69.2% (18) said they were likely to use it for that purpose. See also Table 2.

When the participants who were still smoking were asked what they would be most likely to do if ST were available on the market in Australia, only 1 of them said he would not use it, 3 did not know and 1 failed to answer. Of the other 23, 3 reported possibly using it but continuing to smoke, 9 possibly using it to quit and 10 participants said they would use them to quit and only continue if they couldn't quit without it, and only one participant said he would use them instead of smoking in long-term.

Like in the UK there was no systematic change in beliefs about the harmfulness of the products or of overall likelihood of future use.

It is notable that 45% (14/31) reported either being quit or planning to in the next month after product use suggesting that participating in studies of this kind did not inhibit quitting.

Support for laws reducing cigarette availability (asked at end of questionnaire) was quite strong with 51.7% (15/29) responding that they probably or definitely would support such laws, and only 24.1% (7) opposing (see Table 2). When asked if they would be more likely to support the law if substitutes were widely available none of those opposed (0/7) changed their opinions at all, whilst 15/18 (83%) of those without an opinion or who were probably supportive became more supportive.

#### Qualitative data from Australia

Qualitative data illustrates the diversity of opinion. One participant reported using some of the following three products - nicotine lozenges, Oliver Twist and tobacco tablets - and preferred the lozenges: *"Nicobate CQ lozenge was definitely the best tasting and reduced cravings the most out of the three products I tried."*

Another participant also tried three products (nicotine lozenges, Snus and Oliver Twist) and reported that she used all the nicotine lozenges sent to her. She made the following comments: *"[I] would like to comment on current \'quit\' info campaigns as providing inadequate information. If any of the health professionals I\'ve spoken with re my smoking in the last years had offered me Nicobate lozenges to help me quit I would have been non-smoker."*

But others preferred a smokeless tobacco product. One man commented that: *"teabags were really successful I would consider them for future use. I guess they have changed the way I looked at smokeless products both for satisfaction and cutting the craving." *Another smoker stated that: "*of the three products supplied [Oliver Twist, Snus, tobacco tablets] I found the 'Oliver Twist' one to be the best ... in wanting a cigarette less*...". Another said he used Ariva, and commented that it was working well, that he was just using it like candy, waiting for it to melt, and then using another one.

One participant commented that she found the products good to use and she used all nicotine lozenges and tobacco tablets (another product she tried was Oliver Twist). She was not smoking at the time of the final survey.

It was common to report that while the products did reduce cravings they were not as desirable as cigarettes. For example, one smoker who used some of nicotine lozenges, tobacco bits and tobacco tablets, said that he did not get the same sensation as from smoking, but the cravings subsided.

The major complaint about the products was their taste (eg, too strong, unpleasant taste on the tongue). One participant made the following comments about the products: *"The chewing tobacco leaves a vile solution in my mouth which a swallowed initially and then realised I had to spit it out. It was unpleasant to swallow. I cannot spit it out though as this is more socially unacceptable than smoking in public." *He added, *"as a result I have not quit." *Another who also tried the three smokeless products commented that: *"They tasted horrible! Someone needs to work on the taste. I\'m not sure if I smoke enough for them to be any good to me, cause honestly they made me feel really sick." *Another said: *"I found the \'juice\' from the \'teabags\' to be unpleasant. The tobacco tablets left an unpleasant taste on my tongue."*

It is notable that some reported that the products were too strong. One woman who used some of the nicotine lozenges, Snus and tobacco tablets said that: *"The Tea Bags were too strong for me. I think you need to have your mind ready to quit before you embark on any substitutes - that is half the battle!"*

Some participants shed some light on why they did not try the products sent to them. One participant asked "*why replace one drug with another?" *She said that she did not like the idea of swapping one addiction for another (She tried nicotine lozenges later). Another said that: *" I haven't tried the products yet as I've just started a new job and am very stressed. I will give it a shot when it all settles down and am feeling less stressed"*.

## Discussion

These two preliminary studies indicate considerable interest in using NRT and ST either as a means of quitting smoking or as a long-term substitute for smoking. Taken together and with other similar studies [4-6], they suggest an untapped interest in many smokers' desire to reduce the harmfulness of their smoking using substitutes and provide a range of useful insights for pursuing more definitive research in this area.

The two studies reported here were exploratory and few firm conclusions can be drawn from them (beyond the insights they provide for future research). The samples we report on are of smokers prepared to consider alternatives, and it is likely that attitudes to these products would be much more negative in the general population, especially among those not interested in trying them. However, this may not hold when smokers are interested in quitting, a group excluded from these two studies. The studies are also the first ones done in the two countries, and add to US-based studies especially where the results are concordant.

In interpreting this finding, it needs to be realised that the preferences are ones made after only short term use (less than 1 week) of the products tried, typically with concurrent smoking. We do not know whether the preferences would persist with longer term use or if they tried to use the products initially as part of a quit smoking attempt. However, in Australia at least the findings are similar to a survey of smokers attitudes in the absence of opportunities to use [7], suggesting some stability of interest. The ST market is characterised by lower strength starter products and higher strength products for established users, so escalating to other stronger and potentially more harmful products remains a possibility. However, to get to established use, one needs to start, and it is clear that NRT is at no disadvantage here. Further work is needed to explore implications of longer periods of use and what factors influence sustained use of these products to quit and/or substitute.

Second, the evidence is clear, that the greater the range of options provided, the more likely we are to find one that will be acceptable to any given smoker, thus increasing the potential pool of those who might be helped. However, our studies lack the power to make reasonable estimates of the marginal benefit of adding ST to NRT. In this regard, consideration should be given to including e-cigarettes as part of the mix and to explore whether denicotinised cigarettes in combination with nicotine substitutes enhance the transition away from smoking. Additionally, the strategy employed by O'Connor et al [5] of giving an initial sample pack of products and then getting the participant to choose the product they wanted to use longer term is a sensible and viable approach for encouraging more than minimal use of substitute products.

Third, nicotine replacement products, at least short term hold similar levels of appeal to smokeless tobacco products, perhaps even greater appeal, and any substitute or quit strategy should include them. Whether ST products are needed as well remains unclear, and that is likely to be a function of the range of NRT products that are potentially available. In our studies we only offered one form of NRT and it is likely that adding others would increase the total proportion finding something they were interested in using, and perhaps even reducing the proportion who would prefer a ST product. Further, if other ingredients are allowed in NRT products to increase smoker interest, then they will become increasingly difficult to differentiate for the cleaner forms of ST in potential consumer interest.

While it is possible to conduct studies like this remotely; i.e., without face to face contact, some level of informal contact is desirable to discuss barriers to use and to encourage some to commence use. Overall levels of use of the products appeared to be higher in the UK study involving face to face contact. However, having contact only through the internet or telephone may give a better picture of likely use in real world settings.

These studies were conducted in the context of providing participants with information on the relative harmfulness of various nicotine delivery products. We think it essential that any such work motivated by public health goals does similarly, so that potential participants are properly informed and the potential for misunderstanding the motives of the studies is minimised.

Participation in this study did not act as a barrier to cessation, therefore the precaution we took of not recruiting those planning to quit in the immediate future is probably not warranted, as if it stimulated some quitting in those professing no interest, it seems implausible that it would distract those who were interested at recruitment. Indeed, it seems that encouraging use of substitutes for smoke-delivered nicotine acts as a stimulus for quitting. Although not looked at in this study, it may be the desire to use as a cessation aid that motivates smokers to overcome common mild aversive reactions to the products. If so, we might get higher levels of use among such a group.

If substitutes are to become an accepted part of the tobacco control toolbox, more consideration is needed over their long term future and on the mechanisms by which they are made available. Unless we are content to see an expanding market for substitutes and them becoming a long term part of society, we should not allow for-profit companies to directly market them to consumers [8], rather they should be made available from a not-for-profit source. It should be possible to frame their availability as the lesser of two evils, and that once smoking is eliminated, there will continue to be public education to discourage any use of nicotine, and there should be programs available to help those addicted to the substitutes to quit all nicotine. It is also important to note that the minority of this sample opposed to restricting availability of cigarettes held that view regardless of the availability of alternatives. This suggests that unless more smokers could be convinced of the viability of alternatives, that there is likely to be very strong opposition to restricting cigarette availability from at least a minority of smokers.

On the basis of this research, the government (or NRT providers) should fund further research into the impact of a campaign to persuade smokers who cannot stop to use NRT instead. More research is also needed into the pros and cons of considering ST as part of a harm reduction strategy but this study suggests that ST could play a role, but that an alternative strategy of just offering NRT products for prolonged use is also likely to be feasible, although the total impact may be less than allowing both. This study has not considered other issues, such as impacts of these products on uptake, issues that need to be addressed before adopting a strategy of encouraging either NRT or both NRT and ST as alternatives to smoking. Given the theoretical benefits of a substitution strategy [9], research is needed into what strategies would be required to maximise use of either NRT or both NRT and ST as substitutes for cigarettes, or as aids to quit, while minimising total community use of nicotine products. We need to be constantly aware that if jurisdictions were to take a proactive approach to encouraging Quit or Substitute models, the social dynamics would change and the public would be likely to become better informed about the rationale for this, always assuming the authorities made concerted efforts to ensure the information was available and made efforts to challenge misconceptions.

In conclusion, this study confirms that many smokers are interested in reducing the harmfulness of their smoking behaviour. Smokers deserve to know what the differential risks of potential alternatives are, and to be supported to make the choices that are in their long-term best interests, which is to quit nicotine altogether, but failing that use the least harmful form of nicotine they find acceptable. Governments have a responsibility to ensure the appropriate information is available, and the regulatory framework is in place to facilitate this happening.

## Competing interests

The authors declare that they have no competing interests.

## Authors' contributions

RB initiated the study which KM and AM developed for implementation in the UK, and BK and LL helped RB adapt for Australia and managed the implementation. LL and KM led the data analysis. LL summarised the qualitative findings from Australia. RB developed an initial draft of the factsheet. All authors contributed to the analysis and interpretation of the data and writing of the paper and approved the final manuscript. RB is guarantor of the data.
